# Genome-Wide Analysis of Subependymomas Shows Underlying Chromosomal Copy Number Changes Involving Chromosomes 6, 7, 8 and 14 in a Proportion of Cases

**DOI:** 10.1111/j.1750-3639.2008.00148.x

**Published:** 2008-10

**Authors:** Kathreena M Kurian, David TW Jones, Faye Marsden, Sam WS Openshaw, Danita M Pearson, Koichi Ichimura, V Peter Collins

**Affiliations:** Department of Pathology, Division of Molecular Histopathology, Cambridge UniversityCambridge, UK

**Keywords:** aCGH, array comparative genomic hybridization, ependymoma, microarray, subependymoma, whole genome

## Abstract

Subependymomas (SE) are slow-growing brain tumors that tend to occur within the ventricles of middle-aged and elderly adults. The World Health Organization classifies these tumors within the ependymoma group. Previous limited analysis of this tumor type had not revealed significant underlying cytogenetic abnormalities.

We have used microarray comparative genomic hybridization to study a series of SE (n = 12). A whole-genome array at 0.97-Mb resolution showed copy number abnormalities in five of 12 cases (42%). Two cases (17%) showed regions of loss on chromosome 6. More detailed analysis of all cases using a chromosome 6 tile-path array confirmed the presence of overlapping regions of loss in only these two cases. One of these cases also showed trisomy chromosome 7. Monosomy of chromosome 8 was seen in a further two cases (17%), and a partial loss on chromosome 14 was observed in one additional case.

This is the first array-based, genome-wide study of SE. The observation that five of 12 cases examined (42%) at 0.97-Mb resolution showed chromosomal copy number abnormalities is a novel finding in this tumor type.

## INTRODUCTION

Subependymomas (SE) were first described by Scheinker in 1945 (31). They are slow growing, benign neoplasms of uncertain histogenesis typically located within the ventricles (20). Most cases of SE occur in middle or old age, and they are more frequent in men than women (32). Prognosis is generally good, and surgical removal alone is often curative (19, 24, 27, 28).

The true incidence of these tumors is unclear. In a series of 298 ependymal tumors examined by Schiffer *et al*, they accounted for 8.3% of cases (33). SE occur most frequently within the fourth ventricle (50%–60%), followed by the lateral ventricles (30%–40%) (20). Less common sites include the third ventricle, the septum pellucidum and the spinal cord (16). In the vast majority of cases, they develop sporadically, though very occasional familial cases have been described (5, 6, 13, 30). One report describes infratentorial SE occurring in two identical twins 6).

The proposed precursor cell of the SE is still controversial and suggestions have included subependymal glia (1, 26), astrocytes of the subependymal plate, ependymal cells (21, 29) and a mixture of astrocytes and ependymal cells (4, 10). Recent work has proposed radial glia as the cancer stem cell underlying ependymoma development 10); however, similar studies of SE have not yet been undertaken.

The histological appearance of SE is distinctive, comprised of clusters of glial tumor cells embedded in an abundant fibrillary matrix (20). However, areas with subependymomal morphology can be found in otherwise classical ependymomas (20).

Three previous cytogenetic studies using karyotypic analysis of metaphase spreads looked at a total of six cases of SE. This revealed no cytogenetic abnormalities in five cases, but in one case with a normal karyotype, non-clonal structural abnormalities were identified on the short arm of chromosome 17 (7, 8, 35). A further study using flow cytometry to assess DNA content in 15 cases found aneuploidy in one case of SE, and a higher than normal proportion of cells in G2/M phase in two cases 21). Specific genetic analysis of two SE for allelic deletions on chromosomes 10q and 22q and for point mutations of the NF2 and PTEN tumor suppressor genes did not reveal any changes at these loci (9).

Our aim was to examine a cohort of twelve SE using array comparative genomic hybridization techniques, in order to assess the contribution of copy number change to the oncogenesis of SE. We report two cases with copy number abnormalities on chromosome 6 and two cases with monosomy of chromosome 8, as well as individual incidences of trisomy of chromosome 7 and one partial loss on chromosome 14. To our knowledge, this is the first genome-wide array-based study of SE.

## MATERIALS AND METHODS

### Patients, tumor tissue and DNA isolation

Primary tumor samples from 12 patients with a SE were included in the analysis. The tumors were resected at the Karolinska Hospital, Stockholm, and the Sahlgrenska University Hospital, Gothenburg, Sweden between 1988 and 1997. Full ethical approval has been given for this study. At resection, the median age of the patients was 54 years (range 25–76 years). Patients’ gender, age at resection and tumor location are described in Table 1. Histopathological classification was undertaken according to World Health Organization recommendations. All tumor pieces were selected for DNA extraction after histological examination to ensure a minimum of 80% tumor cells within the samples. DNA was extracted from tumor pieces and blood lymphocytes as described previously (21). Tumor samples were stored at −135°C and blood samples at −20°C before DNA extraction. Extracted DNA was stored at −80°C.

**Table 1:** Results of whole genome microarray comparative genomic hybridization (aCGH).

Case ID	Sex	Age	Location	aCGH result	
				
				Gains	Losses
SE1	M	69	Fourth ventricle		
SE2	M	76	Fourth ventricle		−14q21.1-q31.3
SE3	F	39	Right lateral ventricle		−8
SE4	F	48	Right lateral ventricle		
SE5	M	52	Fourth ventricle		
SE6	F	49	Intramedullary		Complex −6p/6q
SE7	M	52	Brainstem		
SE8	M	56	Brainstem		
SE9	M	25	Intraventricular		
SE10	M	68	Fourth ventricle		
SE11	M	56	Fourth ventricle	+7	−6q13-q15
SE12	M	58	Intraventricular		−8

### Microarray comparative genomic hybridization

A 0.97-Mb resolution whole genome microarray was constructed with 3038 clones obtained from the Wellcome Trust Sanger Institute as described previously (9, 21). The chromosome 6 tile-path array contains 1780 clones [778 P1-derived artificial chromosomes and 1002 bacterial artificial chromosomes (BACs)] that cover 98.3% of the published chromosome 6 sequence. Construction of this tile-path array has been described previously (14).

All labeling and hybridizations were performed as described previously (15, 18). Briefly, 400–800 ng of test and reference DNA were labeled using a Bioprime Labeling Kit (Invitrogen, Carlsbad, CA) with a modified dNTP reaction mixture. Test DNA was hybridized with sex-mismatched reference DNA from samples of pooled blood from 20 normal men or 20 normal women. The labeled and purified DNA was co-precipitated with 45 μg Cot1 DNA (Roche Diagnostic, Mannheim, Germany). The precipitated DNA was dissolved in hybridization buffer, incubated at 37°C for 2 h, and hybridized to the array that had been pre-hybridized with 480 mg herring sperm DNA (Sigma-Aldrich, St. Louis, MO) and 80 μg Cot1 DNA. Arrays were allowed to hybridize for up to 24 h at 37°C and then washed and analyzed as previously described (15, 18). Scanning and analysis of the arrays, criteria for exclusion of spots and scoring of copy number have also been described previously (21).

## RESULTS

Clinicopathological data for the samples as well as any changes seen on the whole-genome array are summarized in Table 1. SE are more common in men than women, and this is reflected by this series (9 men : 3 women). The median age at operation was 54 years. The majority of the tumors (9/12, 75%) were intraventricular, though individual cases located in the brainstem and spinal cord were also included.

Most of the cases examined (7/12, 58%) showed normal copy number across the genome at 0.97-Mb resolution. Five of 12 (42%) cases showed abnormal copy number as shown in Table 1. SE3 and SE12 showed loss of an entire chromosome 8, SE6 showed a complex pattern of losses on chromosome 6 described in more detail below, SE11 showed a gain of chromosome 7 and a partial loss on chromosome 6 (−6q13-q15; maximally between RP3-424L16 to RP1-23D17) and SE2 showed a partial loss of 14q21.1-q31 shown in more detail below.

**Figure 1:**
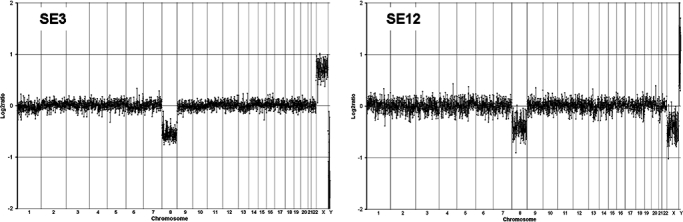
Whole genome microarray comparative genomic hybridization of subependymoma (SE)3 and SE12 showing monosomy 8.

Two copy number alterations were seen in more than one case each. Firstly, both SE3 and SE12 showed loss of one copy of chromosome 8. Whole genome plots of SE3 and SE12 are shown in Figure 1.

**Figure 2:**
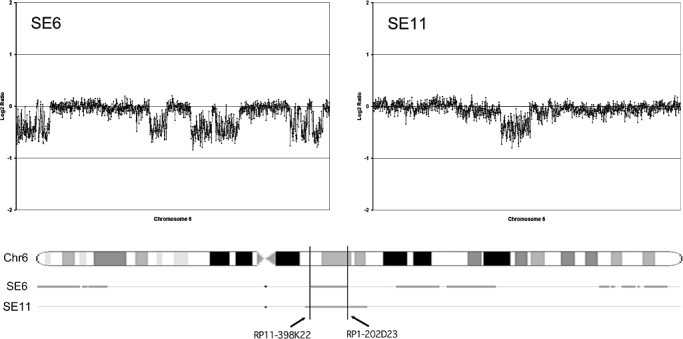
Chromosomal 6 tiling path array on subependymoma (SE)6 and SE11 showing the area of overlapping loss corresponding to the region of loss seen on the 0.97 Mb resolution plot.

In addition, two cases (SE6 and SE11) showed small lengths of overlapping copy number loss within chromosome 6 on the whole genome array. As a result of identification of these apparently relatively small deletions, all cases were subjected to further analysis at higher resolution on a chromosome 6 tiling-path array. The minimal overlapping area of loss in SE6 and SE11 is a 10.01-Mb region between BACs RP11-398K22 and RP1-202D23. The tiling-array plots from SE6 and SE11, along with a schematic representation of the region of overlap are shown in Figure 2. The remaining 10 cases were also examined with the chromosome 6 tiling-path array and no further changes in this region were identified.

**Figure 3:**
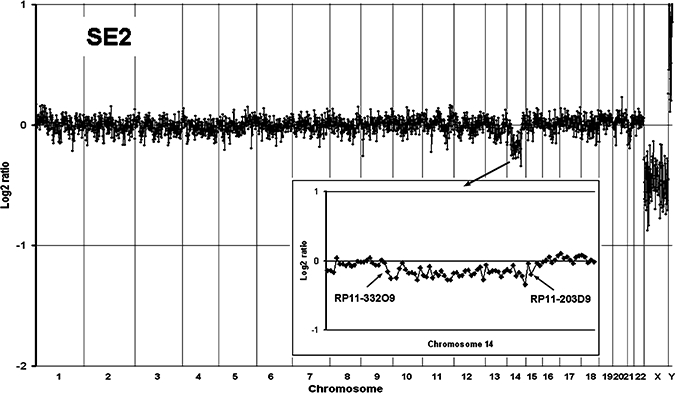
Whole genome microarray comparative genomic hybridization plot of DNA copy number ratio for sample subependymoma (SE)2 showing partial loss of chromosome 14. The inset shows the area of loss and the clones at either end.

SE2 showed a loss of 14q21.1-q31 (maximally between RP11-33209 to RP11-203D9) which is shown in Figure 3.

Chromosomal copy number change was present in 2/3 of the female cases compared with 3/9 male cases, but this was not a significant difference (*P* = 0.52, two-tailed Fisher's exact test). There was no significant difference between the median age of the patients with copy number change (56 years, n = 5) and those without copy number change (52 years, n = 7). There was also no relationship between tumor site and copy number alteration.

## DISCUSSION

This study shows that most of the SE [7 of 12 cases (58%)] show normal chromosomal copy number profiles at this resolution, in keeping with previous work using traditional cytogenetic analysis (7, 10, 36). However, this study shows for the first time that a significant subset of SE [5 of 12 (42%)] show abnormal copy number profiles.

The copy number changes identified include partial loss of chromosomes 6 and 14, trisomy 7 and monosomy 8. In particular, two cases showed overlapping regions of loss on chromosome 6q. Tile-path array analysis confirmed that the area in common is present within two cases only (SE6 and SE11) and is a 10.01-Mb region between BACs RP11-398K22 and RP1-202D23. This region contains 40 gene entries in the Ensembl database which are given in Table S1. Of the forty entries, HMGN3 and TTK tyrosine kinase are candidates which could have tumor suppressor functions. HMGN3 is a nucleosome-binding protein that has roles in chromatin unfolding and transcriptional control (22, 34). TTK, also known as MPS1, belongs to a family of enzymes which can phosphorylate both serine/threonine and tyrosine residues and is involved in the spindle assembly checkpoint (37, 38).

The other findings such as monosomy 8 and trisomy of chromosome 7 have been described in many different tumors. Monosomy 8 has been described in conjunction with other genetic abnormalities in prostatic adenocarcinoma (12, 25). Trisomy 7 has also been reported in peritumoural, non-neoplastic tissues and cell cultures from normal brain (2, 11, 39), and it has been suggested that gain of chromosome 7 in neoplastic and non-neoplastic tissues may be an aging phenomenon (3, 17).

The cytogenetic findings in our study are quite distinct from those commonly found in certain other tumors within the ependymoma group: for example loss of 22q within spinal ependymomas (35) and gain of chromosomes 9 and 18 within myxopapillary ependymomas (23). None of the tumors examined in this study showed a mixed SE/ependymoma morphology.

We show for the first time that relatively large genetic abnormalities occur within SE and indicate that further studies at higher resolution are appropriate to elucidate the cellular processes involved in the development of these tumors.

## Supplementary material

The following supplementary material is available for this article:

**Table S1.** Candidate genes within areas of overlapping of copy number loss.

This material is available as part of the online article from:

http://www.blackwellsynergy.com
