# Supplementary material for: Genome-Wide Analysis of Subependymomas Shows Underlying Chromosomal Copy Number Changes Involving Chromosomes 6, 7, 8 and 14 in a Proportion of Cases
Source: Brain Pathol. 2008 Oct;18(4):469–73. doi: 10.1111/j.1750-3639.2008.00148.x (PMC2659379; doi:10.1111/j.1750-3639.2008.00148.x)
Supplement: Supplementary file 1 [file bpa0018-0469-SD1.doc]

Supplementary table 1
